# Exercise-induced metabolic fluctuations influence AMPK, p38-MAPK and CaMKII phosphorylation in human skeletal muscle

**DOI:** 10.14814/phy2.12462

**Published:** 2015-09-09

**Authors:** Adrien Combes, Jeanne Dekerle, Nick Webborn, Peter Watt, Valérie Bougault, Frédéric N Daussin

**Affiliations:** 1 URePSSS: Physical Activity – Muscle – Health Research Team, EA 7369, University of LilleLille, France; 2 Centre for Sport Exercise Science and Medicine (SESAME), University of BrightonEastbourne, UK

**Keywords:** AMPK, CaMKII, exercise modality, mitochondrial biogenesis, p38-MAPK

## Abstract

During transition from rest to exercise, metabolic reaction rates increase substantially to sustain intracellular ATP use. These metabolic demands activate several kinases that initiate signal transduction pathways which modulate transcriptional regulation of mitochondrial biogenesis. The purpose of this study was to determine whether metabolic fluctuations per se affect the signaling cascades known to regulate peroxisome proliferator-activated receptor *γ* coactivator-1*α* (PGC-1*α*). On two separate occasions, nine men performed a continuous (30-min) and an intermittent exercise (30 × 1-min intervals separated by 1-min of recovery) at 70% of 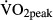. Skeletal muscle biopsies from the *vastus lateralis* were taken at rest and at +0 h and +3 h after each exercise. Metabolic fluctuations that correspond to exercise-induced variation in metabolic rates were determined by analysis of VO_2_ responses. During intermittent exercise metabolic fluctuations were 2.8-fold higher despite identical total work done to continuous exercise (317 ± 41 vs. 312 ± 56 kJ after intermittent and continuous exercise, respectively). Increased phosphorylation of AMP-activated protein kinase (AMPK) (˜2.9-fold, *P* < 0.01), calcium/calmodulin-dependent protein kinase II (CaMKII) (˜2.7-fold, *P* < 0.01) and p38-mitogen-activated protein kinase (MAPK) (˜4.2-fold, *P* < 0.01) occurred immediately in both exercises and to a greater extent after the intermittent exercise (condition *x* time interaction, *P* < 0.05). A single bout of intermittent exercise induces a greater activation of these signaling pathways regulating PGC-1*α* when compared to a single bout of continuous exercise of matched work and intensity. Chronic adaptations to exercise on mitochondria biogenesis are yet to be investigated.

## Introduction

One of the most pronounced effect of endurance exercise is an increase in skeletal muscle mitochondrial content as well as enzymes involved in energy supply (Saleem et al. 2014). These adaptations lead to an enhancement of the muscle aerobic function directly associated with improvement in endurance performance (Daussin et al. 2008) and reductions in risk factors associated with a variety of chronic diseases (Wisløff et al. 2005). At a muscular level, peroxisome proliferator-activated receptor-*γ* coactivator-1*α* (PGC-1*α*) is recognized as a critical regulator of oxidative metabolism, acting as a transcriptional coactivator that regulates adaptive mitochondrial responses to the oxidative state of the cells (Kelly and Scarpulla 2004). The activation of AMP-activated protein kinase (AMPK), p38-mitogen-activated protein kinase (MAPK) and calcium/calmodulin-dependent protein kinase (CaMKII) signaling pathways are well-characterized upstream modulators of PGC-1*α* expression in skeletal muscle (Coffey and Hawley 2007; Jager et al. 2007). These cascades activate downstream regulatory factors (Liu et al. 2005; Thomson et al. 2008), and in the case of AMPK (Jager et al. 2007) and p38-MAPK (Puigserver et al. 2001), also directly phosphorylate PGC-1*α*, thereby increasing transcriptional activation of the PGC-1*α* promoter through auto-regulatory mechanisms. While exercise upregulates PGC-1*α* content (Mathai et al. 2008; Egan et al. 2010), the contribution of exercise characteristics on the upstream signaling cascades has not been fully elucidated. The interplay between intensity, duration, volume and mode of exercise is yet to be understood in such a way that it might inform exercise prescription.

During transitions from rest to exercise in intermittent exercise, metabolic reaction rate may increase substantially as an attempt to maintain the ATP:ADP ratio in the working muscle cells (Kunz 2001). At submaximal intensities, anaerobic glycolysis in the cytosol, and oxidative phosphorylation in the mitochondria provide most of the ATP used during the on-transient phase. These various reactions cause metabolic disturbances within the cell so that the succession of on-transient phases during intermittent exercise induces a repetition of metabolic changes, defined here as metabolic fluctuations, that are thought to promote mitochondrial biogenesis. Such metabolic fluctuations alongside exercise intensity (Egan et al. 2010) could explain why high-intensity, low-volume interval training may induce similar muscular adaptations to those observed after a more traditional low-intensity but high-volume continuous training, even when the interval training sessions were short (Gibala et al. 2006; Burgomaster et al. 2008). Different training programs have been compared to determine the influence of metabolic fluctuations on aerobic function (Mohr et al. 2007; Edge et al. 2013). However, the results are controversial. For instance, greater skeletal muscle adaptations have been observed following high-intensity exercise training regimen that induces greater metabolic fluctuations (Mohr et al. 2007). Increasing the duration of the rest period between identical exercise bouts attenuates the metabolic fluctuations and the metabolic consequences, in terms of H^+^ and other metabolites, were lessened. Despite this, the consequences, maximal oxygen consumption, and training effects were not different with either a short or longer rest period during interval training (Edge et al. 2013). Whether the metabolic fluctuations *per se* could activate in itself the signaling cascades leading to PGC-1*α* is unknown.

In order to identify whether metabolic fluctuations *per se* are involved differently to those observed in continuous exercise in the signaling pathways for mitochondrial biogenesis, we compared an intermittent and a continuous exercise performed at same exercise intensity for it not to be a confounding factor. We hypothesized that for greater metabolic fluctuations during the intermittent exercise there would be greater increases in stimulatory factors regulating mitochondrial biosynthesis, that is, AMPK, CaMKII, and p38-MAPK phosphorylation postexercise.

## Methods

### Participants and ethical approval

Nine healthy active men participated in this study (Age: 22 ± 5 years; Mass: 74 ± 11 kg; Height: 1.79 ± 0.04 m; 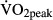: 44 ± 6 mL · min^−1^·kg^−1^; WR_peak_: 261 ± 22 watts). The volunteers were instructed to pursue their habitual training throughout the study, and to refrain from alcohol and caffeine intake for at least 48 h prior to any of the testing sessions. All the subjects provided signed informed consent prior to their participation. Protocol was approved by the University of Brighton Ethics Committee and conducted according to the Declaration of Helsinki.

### Preexperimental procedures

Seven days before the first experimental trial, all participants performed an incremental exercise test to exhaustion on an electrically braked cycle (Schoberer Rad Messtechnik with 8 strain gauges, SRM, Germany) to determine 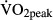 and peak work rate (WRpeak). The test began with a 3-min stage at 75 watts followed by increments of 25 watts every 2 min until volitional exhaustion. Each subject carried out a maximal effort, according to the criteria of Howley et al. (1995).

### Experimental trials

Subjects were required to complete two work-matched acute exercise trials on separate occasions in a random order separated by 1 week. Both exercises consisted of 30 min of active cycling at 70% of WR_peak_ in a continuous (CON) or intermittent (INT) modality. The intermittent exercise was constituted of 30 periods of 1-min work intercepted with 1-min of recovery. Subjects were asked to maintain a pedaling frequency of 75 revolutions per minute and they were instructed to reproduce the same diet for 24 h prior each trial.

On the day of each trial, subjects arrived at the laboratory in the morning, 60–90 min after ingesting their habitual breakfast. A resting muscle biopsy sample was obtained from the *vastus lateralis*, immediately frozen in liquid nitrogen, and stored at −80°C until further analysis. After a 10-min resting period, subjects completed a standardized 10-min warm-up at 40% WR_peak_ followed by a 5-min resting period. Then, participants performed the designated exercise modality. Muscle biopsy samples were obtained immediately upon cessation of cycling and 3 h post exercise. Subjects rested quietly in the laboratory until the last biopsy, and were allowed to consume only water ad libitum.

### Apparatus and analysis

Power output and pedaling frequency were continuously recorded using an SRM recording device (SRM Power Control V). Estimation of the total work done for each exercise was determined and expressed in kilojoules (kJ). Heart rate (HR) was measured continuously during each exercise (RS 800, Polar, Kempele, Finland). Pulmonary gas exchanges were computed breath-by-breath using an on-line gas analysis system (MediSoft, Bochum, Germany). Outliers values were removed as per Lamarra et al. (1987) and signal was interpolated to 1-sec intervals, and averaged over 5-sec periods. 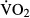 was used to determine the following variables: the accumulated O_2_ consumed over the exercise period (L) and the 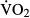 Fluctuations Index (OFI) computed from the two variables characterizing the 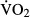 fluctuation (amplitude and rate of rise, see eq. 1).

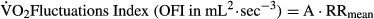
1where A is the sum of 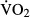 gains in L · min^−1^ and RR_mean_ is the mean 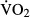 rate of rise (+d
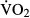
·dt^−1^) in mL· sec^−2^ during exercise.

### Muscle biopsies

Each muscle biopsy sample was taken from the muscle *vastus lateralis* under sterile conditions and local anesthesia. An area of skin and the underlying tissues was anaesthetized with 1 mL of 2% lidocaine and a small (0.5 cm) incision made in order to obtain a tissue sample. A fresh incision was made for each of the three biopsies, in the same leg, at least 2 cm from a previous biopsy site.

### Western blot

**Figure 1:**
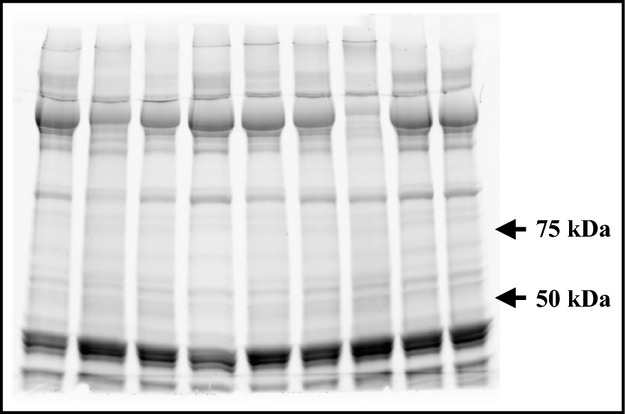
Representative staining gel used as loading control, achieved with entire lanes.

**Figure 2:**
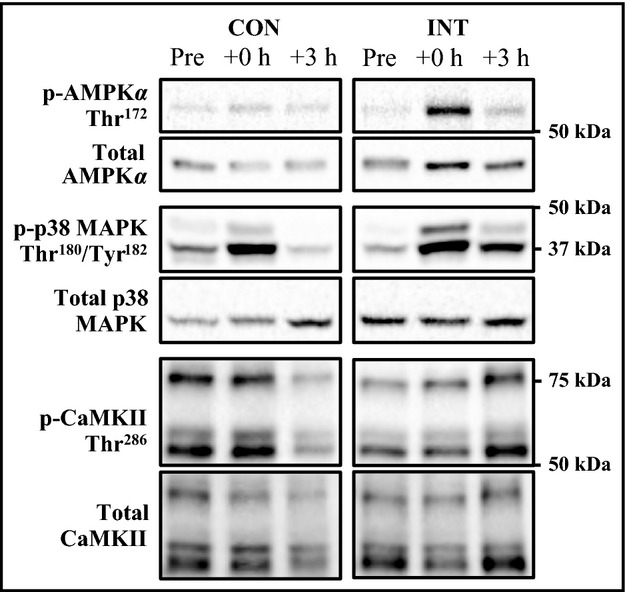
Representative immunoblots corresponding to phosphorylated and total protein expression measured before (Pre), immediately after (+0 h) and after 3 h (+3 h) of recovery from the CON and INT exercise.

The frozen muscle samples were homogenized and proteins were separated using a 7.5% mini precast gels (Mini-PROTEAN TGX Stain-Free, Biorad, Hercules, CA) for 40-min at 200V constant voltage (Mini-PROTEAN Tetra cell and PowerPac Basic power supply, Hercules, CA). Each subject modality samples (continuous or intermittent and Pre, +0 h and +3 h after exercise) were loaded together on the same blot. After the electrophoresis, the total protein quantification as reference was determined with the staining of the gel (Aldridge et al. 2008; Welinder and Ekblad 2011), achieved with entire lanes. Gel proteins were transferred to a 0.2 *μ*m nitrocellulose with a transfer pack (Trans-Blot Turbo, Bio-rad, Hercules, CA) for 10 min at a constant current of 2.5A with an upper limit of 25V. Non-specific binding was blocked in a 5% milk/TBS-t (10 mmol L^−1^ Tris pH 7.5, 100 mmol L^−1^ NaCl, 0.1% Tween 20) for 2 h at room temperature. Membranes were incubated overnight with a primary antibodies: phospho-AMPK*α* Thr172 (#2531), AMPK*α* 23A3 (#2603), phospho-CaMKII Thr286 (#3361), CaMKII pan (#3362), phospho-p38 MAP Kinase Thr180/Tyr182 (#9211) and p38-MAPK (#9212) (1:1000; Cell Signaling Technology, Beverly, MA). Membranes were washed in TBS-t and incubated with appropriate secondary horseradish peroxidase-conjugated antibodies (1:3000; Cell Signaling Technology), visualized by enhanced chemiluminescence (ECL; GE Healthcare, Arlington Heights, IL) and quantified by densitometry (GS800 Calibrated Imaging Densitometer, Bio-Rad, Hercules, CA). Total protein quantification and total protein abundance of each phosphorylated protein were used for normalization where appropriate. A representative staining gel and blot for each protein analyzed are presented in Figures1 and 2, respectively.

### Statistical analysis

Data are presented as means ± SD. Statistical analyses were performed using Sigma Stat for Windows (version 3.0, SPSS Inc., Chicago, IL). After testing for normality and variance homogeneity, a two-way Analysis of variance with repeated measures was used to identify differences in the activation of signaling cascades. When a significant interaction was detected, data was subsequently analyzed using a post hoc Tukey test. To compare the total work done and all variables associated with the 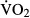 measurements between the two exercises, a one-way ANOVA was used and followed with a post hoc Tukey test. The significance level was set at a *P *< 0.05.

## Results

### Energy expenditure and 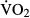 responses

No significant difference was observed between the overall accumulated O_2_ consumption throughout the two exercises (in L: 87 ± 11 and 86 ± 17, for INT and CON, respectively), and the total amount of work accumulated (in kJ: 317 ± 41, and 312 ± 56, for INT and CON, respectively). The 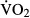 and HR, mean and peak percentage, were presented in Table 1. The representative 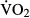 responses during intermittent exercise showed systematic oscillations in synchrony with the work to rest duty-cycle over the exercise duration. Both peaks and nadirs of the 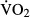 oscillations increased slightly over the first cycles until the values remained unchanged. This amplitude remained constant from the second repetition. The 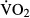 response analysis reveals a 2.8-fold higher metabolic fluctuation for the intermittent exercise (in mLO_2_^2^ · sec^−3^: 9620 ± 2285 and 3723 ± 1021, for INT and CON, respectively).

**Table 1:** Mean and percentage peak of 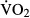 and HR

	CON	INT
VO_2_ (L/min)		
Mean	2.627 ± 0.31	1.443 ± 0.18
% Peak	80 ± 8	45 ± 8
HR (bpm)		
Mean	155 ± 16	120 ± 15
% Peak	87 ± 11	68 ± 11

Mean and percentage peak of 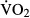 and HR for continuous (CON) and intermittent (INT) trails.

Values are means ± SD.

### Signaling proteins

**Figure 3:**
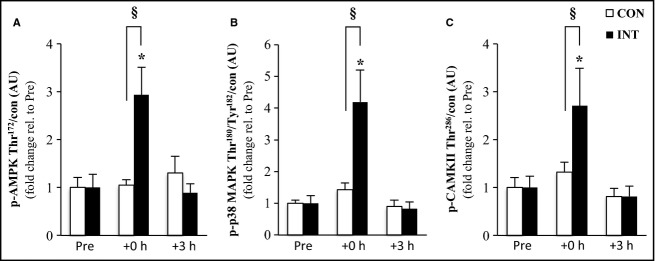
Activation of signaling kinases. The effect of exercise modality on phosphorylation of AMPK (A), p38-MAPK (B) and CaMKII (C) protein immediately after (+0 h) and during recovery (+3 h) from isocaloric exercises. Open bars represent continuous trial, CON; filled bars represent intermittent trial, INT. Phosphorylated protein is normalized to total protein content (con) of the respective protein. Values are means ± SEM., *n* = 9. *Significantly different from baseline within same trial (*P* < 0.01); §significantly different from CON at the same time point (*P* < 0.05).

AMPK, CaMKII, and p38-MAPK phosphorylation were increased ˜2.9, 2.7 and ˜4.2-fold, respectively, immediately after exercise (+0 h) following the intermittent (*P* < 0.01) but not continuous exercise, neither group showed increased phosphorylation 3 h after exercise finished (Fig. 3). The phosphorylation of the three kinases increased to a greater extent (˜2.1, 2.9 and ˜2.8-fold, respectively, for APMK, CaMKII, and p38-MAPK) following the intermittent exercise compared to the continuous exercise (condition-by-time interaction, *P* < 0.05).

## Discussion

We examined changes in signaling protein phosphorylation in human skeletal muscle after two different exercise modalities (continuous vs. intermittent). For the same intensity and duration of cycling, and therefore work done, the intermittent exercise-induced repeated metabolic fluctuations and higher phosphorylation of AMPK, CaMKII, and p38-MAPK compared with the continuous exercise. These kinases are three important signaling cascades linked to PGC-1*α* and the regulation of mitochondrial biogenesis in skeletal muscle. These results suggest that for a same exercise intensity and work done, activation of signaling pathways involved in mitochondrial biogenesis would be greater following intermittent training.

### Metabolic fluctuations

It is during both the on- and off-transient of each bout of intermittent exercise that metabolic disturbances occur and reaction rates change rapidly. Using a physiology-based computational model of skeletal muscle energy metabolism, Li et al. (2009) quantified the key regulatory factors of skeletal muscle energy metabolism during transition from rest to exercise at various exercise intensities. At 60% of 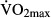, the cytosolic redox state (NADH/NAD^+^) increases to reach a peak 2-min after the beginning of exercise to then decrease progressively. A concomitant 50% decrease in the ATP:ADP ratio was observed at the beginning of exercise, to then increase progressively and stabilize to 75% of resting values after 15 min of exercise. This model suggests that cellular homeostasis is clearly most perturbed in the first few minutes of exercise. Therefore, the repetition of 1-minute work/rest periods during the intermittent exercise would induce a succession of metabolic oscillations that trigger protein phosphorylation. Interestingly in the present study, the metabolic fluctuations during the intermittent exercise were ˜3 fold those of the continuous exercise, the same extent in scale (from 2.8 to 4.2-fold) to the change in kinases phosphorylation observed post exercise.

### Signaling pathways activation

Most of the studies on kinases’ acute regulation related to mitochondrial biogenesis have used very prolonged exercise interventions. In mice, Malek et al. (2013) compared a 30-min continuous exercise to 3 bouts of 10-min, separated by 2 h, at the same intensity and found a similar phosphorylation of p38-MAPK, and protein expression of MEF2A and PGC-1*α* in each exercise. However, the small number of repetitions and the large rest period between them would have led to insufficient metabolic fluctuations and/or overcompensation for a difference in kinase activation to be observed (Braun and Schulman 1995). In our study, both exercise regimes lead to similar levels of phosphorylation after 3 h, indicating some down regulation or dephosphorylation events during the rest period. In humans, Cochran et al. (2014) compared four 30-sec Wingate tests interspersed with 4 min of rest to a work-matched continuous high-intensity exercise of 4 min and found similar increases in AMPK and p38-MAPK activation in each exercise. Mean power output during the intermittent exercise was about twice that of the continuous exercise so greater changes might have been expected in their experiment if intensity was the sole regulator of AMPK and p38-MAPK phosphorylation (Edgett et al. 2013). However, the number of repetitions, and therefore occurrence of metabolic fluctuation at the cellular level, despite their severity, was likely to be fairly low. This may explain the lack of difference observed between the two conditions. Bartlett et al. (2012) compared two matched exercises (6 × 3-min at 90% 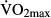, vs. 50-min at 70% 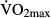) that elicited similar increases in AMPK and p38 MAPK activation probably due to too similar stimulus and/or a low number of repetitions (i.e. fluctuations). Taken together, our results suggest that for a given exercise intensity, metabolic fluctuations caused by the succession of on- and off-transients during an intermittent exercise are critical to maximize activation of the PGC-1a signaling pathway in skeletal muscle.

### Overcompensation of kinase activity

#### AMPK mechanisms

Muscular activity leads to an increase in the AMP:ATP ratio. AMP binds to the AMPK cystathionine-b-synthase (CBS) domain of the *γ* subunit, to activate AMPK (Davies et al. 1995; Suter et al. 2006). Recently, Xiao et al. (2011) showed that ADP can also bind to one of the two exchangeable AXP-binding sites on the AMPK regulatory domain and also protects the enzyme from dephosphorylation. These studies showed that active AMPK displays significantly tighter binding for ADP and AMP than ATP, explaining how AMPK may be regulated under physiological conditions, where the concentration of ATP is higher than that of ADP and much higher than that of AMP. Therefore, an increase in ADP may be the primary signal that promotes increased phosphorylation of AMPK, especially during moderate energy stress. In the context of our intermittent exercise and considering that ADP concentration increases transiently at the beginning of exercise (Li et al. 2011), a repetition of on-transient phases may induce greater variations in the AMP:ATP ratio when compared to the continuous exercise. This is in support of the assumption that metabolic fluctuations may potentiate the AMPK phosphorylation during exercise, thus independently of exercise intensity.

#### CaMKII mechanisms

During muscle contraction, Ca^2+^ release binds to and activates the regulatory protein CaM. CaM then binds to the CaM-binding domain of CaMKII leading to its conformational change by dissociation of the auto-inhibitory domain from the catalytic domain. Furthermore, prolonged activation of CaMKII by the interaction of CaMKII with CaM results in the autophosphorylation of threonine-286 (T286) (Kato et al. 2013). Phosphorylation at T286 is thought to stabilize the CaM-bound form of CaMKII and therefore, prevents the inactivation of CaMKII kinase activity even after the dissociation of CaM. This maintains its activity (Braun and Schulman 1995; Singla et al. 2001) so that substrates, like PGC-1*α*, can be phosphorylated. With periods of increases and then decreases in [Ca^2+^] outside the sarcoplasmic reticulum, a larger number of CaMKII subunits are likely to be activated so that the above-mentioned complex has greater Ca^2+^-independent CaMKII activity (Chin 2005). These reactions are likely to be greater during the intermittent exercise carried out in the present study. The activity of CaMKII is probably maintained, giving more time for kinase activity but also allowing for a greater recruitment of CaMKII kinases. Despite a similar quantity of work in both exercises, the kinase seems sensitive to the pattern of the exercise. Similar autophosphorylation mechanisms have been also observed for AMPK (Woods et al. 2003) and p38-MAPK (Kim et al. 2005) suggesting that the intermittent pattern of the exercise *per se* would enhance these kinases activity similarly to CaMKII.

#### p38 MAPK mechanisms

The p38-MAPK, sensitive to mechanical stress, is also involved in mitochondrial biogenesis as it can directly phosphorylate PGC-1*α* (Puigserver et al. 2001). Despite a similar total amount of work accumulated during the two exercises, we observed a higher p38-MAPK phosphorylation following the intermittent exercise. When raising cytosolic Ca^2+^, Wright et al. (2007) observed an increase in p38-MAPK phosphorylation suggesting that p38-MAPK is a downstream of CamKII. We can speculate that this pathway is also activated during exercise for the greater activation of CaMKII observed after the intermittent exercise to explain the greater p38-MAPK phosphorylation.

### Perspectives and significance

We are unable to perform PGC1*α* mRNA measurements, which would have potentially resolved the question is whether the divergent response observed between the continuous and intermittent exercise protocols translated into differences in PGC-1*α* expression. The observed molecular responses were observed in a specific population. As such, studies with similar measurements and PGC1*α* mRNA in deconditioned individuals as well as highly trained athletes are much warranted to further improve our understanding in how exercise modality may be used to enhance muscle oxidative adaptations to exercise.

In conclusion, the reoccurrence of metabolic fluctuations induced by an intermittent exercise appears to be a potent stimulus for upstream kinases activations, which are recognized as modulators of PGC-1*α*. The intermittent modality would be “metabolically more effective” at stimulating the signaling pathways leading to mitochondrial biogenesis. Futures studies should investigate the effect of exercise modality on PGC-1*α* and mitochondrial biogenesis in acute and chronic exercise for the all-signalling pathways to be better understood.
